# Special Issue “New Molecular Mechanisms and Advanced Therapies for Solid Tumors”

**DOI:** 10.3390/ijms27062520

**Published:** 2026-03-10

**Authors:** Valentina De Falco

**Affiliations:** 1 Institute of Endotypes in Oncology, Metabolism and Immunology “Gaetano Salvatore”, Department of Biomedical Sciences, National Research Council (CNR), 80131 Naples, Italy; valentina.defalco@cnr.it; 2 Faculty of Medicine, UniCamillus-Saint Camillus International University of Health and Medical Sciences, 00131 Rome, Italy

Solid tumor research is undergoing a decisive transition: innovation is no longer simply “finding a new receptor” or “inhibiting a new enzyme”, but integrating targets, biomarkers, and therapeutic modalities into strategies capable of anticipating tumor adaptation and therefore mechanisms of resistance. It is necessary to develop new integrated resources, with a 360-degree view of the disease, that are flexible over time and adaptable to changing patient needs. Tumors escape linear blocks, activate bypass pathways, remodel the microenvironment, and shift their metabolic and immunological state; for this reason, today the notion of therapeutic target encompasses nodes of proteostasis and the epigenome, emerging immune checkpoints, surface targets exploitable by antibodies and antibody–drug conjugates (ADCs), metabolic vulnerabilities, and invasion/metastasis programs governed by the stromal ecosystem.

This Special Issue aims to collect works that offer exemplary perspectives on emerging topics useful for properly addressing new therapeutic approaches. The collection brings together contributions that converge on one point: connecting basic biology and clinical translation, defining “who to treat,” “with what,” and “how to combine” to maximize benefit and duration of response.

The first axis emerges from the work of Kreienbühl and colleagues (Contribution 1), who show that the ubiquitin ligase CUL4B is not a simple redundant paralogue of CUL4A but is a critical node of plural mesothelioma: its reduction impairs clonogenicity and proliferation and increases cell death, but above all it impacts programs linked to the Hippo pathway (YAP1, CTGF, survivin) and, selectively, to TGF-β signaling. The finding that downregulation of CUL4B reduces TGF-β1 and MMP2 expression directly links it to pro-fibrotic programs, matrix remodeling, and cell invasion, properties that fuel aggressiveness and refractoriness to mesothelioma treatments. The authors further discuss inhibition of neddylation with pevonedistat as an approach to interfere with cullin function, reporting that the effect observed in vivo—increased cell death and reduced TGF-β1/MMP2 in tumor tissue—may depend on microenvironmental components (hypoxia, stroma, immunity) absent in in vitro systems. This divergence between in vitro and in vivo models is a cross-cutting message on how to evaluate new targets as some vulnerabilities become visible only in the tumor ecosystem. Looking ahead, this strand engages with the recent expansion of targeted protein degradation (TPD), which exploits the ubiquitin–proteasome system (and, in some variants, lysosomal pathways) to selectively eliminate pathogenic proteins through PROTACs and molecular glues [1]. Altering protein homeostasis by completely eliminating a target protein offers the possibility of reducing resistance phenomena dependent on mutations or overexpression of the protein itself. For proteins that are not easily inhibited, this approach proves to be very advantageous.

Another possibility is to expand immunotherapeutic options beyond PD-1/PDL-1. In the work of Kula and colleagues (Contribution 2), the immunosuppressive role of HHLA2, a protein of the B7 family often overexpressed in various solid tumors, is described. In their work, through an integrated analysis involving immunohistochemistry (HHLA2, MSI, CD8+), morphological studies (tumor budding and TILs), cytokine/chemokine profiles, and bioinformatics, they identify the contribution of HHLA2 to immunological evasion. HHLA2 is interpreted as an emerging, potentially bifunctional checkpoint, whose relevance depends on the immunological context and subtype (e.g., MSI vs. MSS). Looking ahead, this contribution advocates for second-generation immunotherapies based on rational drug combinations and immune targets alternative to traditional checkpoints, especially in settings where the response to PD-1/PD-L1 or CTLA-4 is incomplete or transient. The field of “non-canonical” checkpoints is maturing: LAG-3 inhibition with nivolumab–relatlimab shows a long-term survival benefit in advanced melanoma [2], but overall experience indicates that these targets require finer profiling of the immunological context (T cell exhaustion, myeloid compartment, interferon signature, stromal immunosuppression) and dynamic biomarkers. In this logic, HHLA2 should be interpreted as a node of immunoregulatory circuits and not as an isolated switch. Interest is growing in molecules that combine PDL-1 and VEGF in a single drug (for vascular blockade and immunosuppression), with global programs in advanced stages and large pharmaceutical partnerships; this is an example of “mechanism integration” through a single tool. One example is the antibody BNT327, BioNTech’s PD-L1xVEGF-A bispecific antibody [3]. Furthermore, metabolites and microbial composition may influence the response to ICIs and irAEs; various interventions (targeted diet, FMT, modulation trials) and the intratumoral microbiome as an additional level of the TME are being evaluated [4].

A third axis is the quality of biomarkers, a topic addressed by the contributions on breast cancer from Milosevic and colleagues (Contribution 3). They studied mammaglobin as a relatively specific biomarker for breast tissue, useful for diagnosis, monitoring and stratification. The lesson is general: a biomarker becomes truly useful when it improves risk stratification and enables longitudinal follow-up, allowing for more personalised treatment decisions. This requires, in addition to biological plausibility, analytical and clinical validation in independent cohorts and in real-world settings, with a clear definition of its intended use (screening, differential diagnosis, prognosis, or monitoring). Slide-based AI (computational pathology) is used to reduce scoring variability, discover morphological signatures associated with responses, and integrate imaging with omics (a multimodal approach). Industrial interest and R&D initiatives in AI/quantitative pathology are rapidly accelerating [5].

Another topic is addressed by Kurokawa and colleagues (Contribution 4). They show how metabolomics and metabolic profiling can generate predictive biomarkers and open therapeutic vulnerabilities, linking metabolism, plasticity and response to treatments. They apply untargeted plasma metabolomics to distinguish low- and high-grade meningiomas, to anticipate signs of aggressiveness early before complete histological definition. A panel of metabolites associated with high-grade tumors shows high specificity, suggesting that systemic metabolic profiling may reflect the biological status of the tumor. The outlook is promising but challenging: pre-analytical standardization, confounder control, and external validation are mandatory steps for clinical translation and to avoid “fragile” models that do not generalize. These perspectives gain further weight in light of recent studies that have redefined thresholds and significance of surface target expression: in the phase III DESTINY-Breast06 trial, trastuzumab deruxtecan prolonged progression-free survival compared to chemotherapy after endocrine therapy in patients with HR-positive HER2-low and HER2-ultralow metastatic breast cancer [6]. In this case, the biomarker becomes a practical tool for establishing access to treatment and risk management. Treatment efficacy depends not only on biomarker levels but also on the ability of the antibody to selectively release its cytotoxic cargo.

A fifth topic addressed concerns rare but paradigmatic neoplasms, such as Ewing’s sarcoma (Contribution 5). The pediatric disease presenting the EWS/FLI1 fusion, a difficult-to-target driver, is discussed by Yasir and colleagues in their contribution. They emphasize that the driver’s activity, by modifying transcriptional and epigenetic programs, supports plasticity and heterogeneity even in a low-mutation setting. This analysis requires targeting downstream substrates after their identification through chemical/genetic screening. This allows for the identification of effective and synergistic drug combinations that also take into account low toxicity, a crucial point in pediatric age. In terms of targeting, the important target becomes the network, and not only the oncogenic fusion. This means that when the driver is a transcriptional program, it becomes crucial to map the network dependencies and exploit novel modalities (such as targeted degradation) to destabilize complexes and interactions.

A sixth topic concerns metastatic disease and loco-regional therapeutic strategies. In their contribution, Panczel and colleagues (Contribution 6) evaluate the use of cytoreductive surgery combined with HIPEC for the treatment of peritoneal metastases from gastric cancer, highlighting patient selection criteria, limitations of the combined treatment type, and potential benefits. Their analysis shows a survival advantage for the combination, especially in the short term (first two years), consistent with the HIPEC goal of eliminating microscopic residual peritoneal disease. The authors also emphasize the need for standardization and prospective studies to clarify which patients benefit the most, classifying them based on peritoneal involvement, histology, and completeness of cytoreductive surgery. The rationale for undertaking a therapeutic approach must take into account a series of factors that influence efficacy as synergistic combinations between drugs may produce different responses in case of suboptimal choices. Correct management of immunotherapy and targeted therapies with a choice of a systemic or loco-regional approach is essential to prevent or eradicate metastasis. The discovery of a new target in gastric cancer has transformed therapies. This is the surface protein Claudin 18.2 (CLDN18.2), which is inhibitable with the monoclonal antibody zolbetuximab-clzb used in combination with chemotherapy for HER2-negative CLDN18.2-positive patients (FDA, 18 October 2024) [7]. This demonstrates that real progress is made when targets are chosen specifically for individual patients, always considering the possibility of measuring tissue-specific vulnerability in real time.

The seventh axis concerns the ability of tumor cells to adapt, change identity, and become resistant to therapies, along with their capacity to invade and the relationship between tumor and stroma. The discussion on the importance of invasion-related targets and cell–matrix interactions uses prostate cancer as a model. In their article, Santos and colleagues (Contribution 7), through transcriptomic analyses, immunohistochemistry and prognostic studies on publicly available datasets, characterize the SLIT/ROBO axis in both murine models and human samples. Alterations in SLIT/ROBO genes are associated with a higher risk of recurrence, indicating that their mutations are able to predict aggressiveness and risk of progression [8]. This contribution strengthens the idea that, in heterogeneous tumors, markers related to invasion and tumor–stroma crosstalk can be very useful to improve stratification and clear the way for therapeutic targets complementary to antiproliferative strategies, especially when the clinical goal is to distinguish indolent from aggressive forms.

All of the above highlights the need to move beyond simple oncogene analysis to focus on the more general behavior of tumors. Targeting an invasion program or communication axis can be crucial in preventing recurrence and metastasis, especially when combined with tumor-reducing therapies and biomarkers capable of early intercepting the transition to aggressive phenotypes. Some general insights emerge from the contributions to the Special Issue.

First, the target is not static. Expression, localization, and function change over time and under therapeutic pressure; therefore, longitudinal biomarkers (tissue and liquid biopsy) and adaptive decisions are needed. ctDNA/MRD (minimal residual disease) is moving from a prognostic indicator to a postoperative stratification and a potential guide for adjuvant therapy (guidelines on when to change strategy). Recent data on colon cancer (stage III) and numerous clinical trials are consolidating this approach [9].

Second, drug combinations are the rule. The example of anti-KRASG12C therapy in colorectal cancer is instructive: adagrasib plus cetuximab shows greater efficacy in pretreated patients, preventing the known feedback resistance mechanism via EGFR [10]. Third, personalization can become therapy. In the KEYNOTE-942 trial, personalized neoantigen therapy mRNA-4157/V940 in combination with pembrolizumab improved outcomes in resected high-risk melanoma, demonstrating that “tailored” platforms can be evaluated in randomized trials [11]. Furthermore, for metastatic or unresectable melanoma, the approval of lifileucel which uses tumor-infiltrating lymphocytes (TIL) after the use of anti-PD-1 demonstrates that cell therapy is ready to enter into use also in solid tumors, making it urgent to study the management of toxicities and logistical complexities [12].

Fourth, new levels of intervention using cell biology are emerging. The phenomenon of ferroptosis, a form of regulated cell death dependent on iron-dependent lipid peroxidation levels, has also become a new potential target; there is now a need to identify therapeutic windows and biomarkers that will enable clinical application [13]. New forms of regulated death linked to metabolism, copper/redox, and structural fragility are also emerging—especially useful for thinking about therapeutic windows and biomarkers of sensitivity/resistance [14].

From all of the above, some practical considerations arise. First, to validate a target, it is essential to use models that preserve, to the extent possible, the relevant microenvironmental components (stroma, matrix, immunity), as the therapeutic effect can emerge or change depending on that context. Furthermore, candidate biomarkers (molecular, omic, or clinical procedural) that allow for prospective stratification should accompany each targeting proposal to always establish in real time not only “who” can be treated and for how long, but also “when” and “with what” to combine. To improve therapies, it is no longer enough to know whether a target is expressed; it is crucial to know where it is expressed and in which cellular niches (tumor, stroma, immunity). Spatial-omics is becoming a tool for predictive biomarkers and for explaining local resistance [15].

Another noteworthy consideration is that not all targets are directly targetable. Therefore, understanding the entire network of functional interactions is an advantage because it offers alternative attack strategies. Planning drug combinations that limit cell adaptation while minimizing toxicity is desirable. Even when the target is directly attackable, as in the case of CRS/HIPEC, reproducible and comparable therapeutic results depend on clear technical parameters and knowledge of early endpoints. The combination of these procedures determines the real possibility of translating a potentially promising new target into the clinic.

In conclusion, new therapeutic targets in solid tumors are not just novel molecules but actual conceptual maps. This Special Issue emphasizes that the most robust innovation occurs when basic mechanisms (proteostasis, transcription, metabolism, immunity) are linked to measurable biomarkers and modern therapeutic platforms (targeted degradation, ADCs, vaccines, and cell therapies) as well as to local–regional approaches. Starting from PSMA (prostate-specific membrane antigen), targets and combinations are being expanded (with immunotherapy, DDR-inhibitors, etc.), with a very “biomarker-driven” logic: diagnosis and patient selection with imaging + targeted treatment with radionuclides [16].

The next step will be to make these capabilities interoperable: designing mechanism-based combinations, monitoring developments in real time, and intervening adaptively. The long-term success lies in the ability to choose the right target, for the right patient, at the right time. The innovative potential for establishing new therapies also depends on how we test therapies today: platform/adaptive trials, basket/umbrella trials, the use of intermediate endpoints (e.g., MRD/ctDNA), and integration with real-world data are becoming central to anticipating signals of benefit and predicting the development of resistance [17,18].

In this Special Issue, not only have new targets been proposed, but an operational criterion has been designed to validate their use. In this context, the microenvironment in which they are located is inseparable. Simplified systems are limited by the inability to fully characterize the tumor ecosystem (stroma, immunity) and to account for potential conditions such as hypoxia. Preserving the microenvironment and developing biomarkers capable of understanding the context are among the challenges of the future.

Another area that needs to be addressed is better consideration of the temporal dimension, i.e., the fact that the therapeutic target is not static but is subject to changes that also depend on the therapeutic pressure itself. The collected papers highlight the importance of tissue and liquid biopsy biomarkers, which allow us to monitor changes over time until we reach the prospective goal of being able to anticipate an escape trajectory and plan preventive combinations.

Ensuring biomarkers’ robustness to ensure their clinical utility is also an essential objective. An added value of this Special Issue is clarifying the chain of requirements needed to transform a biological signal into something we can concretely rely on. The themes of standardization, controls, independent cohorts, and real-world evidence are fully highlighted in the selected papers, which explicitly explain the potential chain of requirements that can transform a biological signal into a decision-making tool.

Another topic addressed is the possibility of directly inhibiting certain targets. In these cases, the dependency network with blockade of transcriptional/epigenetic programs becomes key, along with targeted protein degradation or proteostasis nodes.

The suggested ultimate goal is to find, through dynamic biomarkers and platforms useful for designing rational drug combinations, an effective personalized therapy that is both flexible and targeted.
